# Performance variables and nutritional status analysis from Moroccan professional and adolescent football players during the competition period: a descriptive study

**DOI:** 10.3389/fspor.2024.1372381

**Published:** 2024-10-28

**Authors:** Mourad Oukheda, Halima Lebrazi, Abdelfettah Derouiche, Anass Kettani, Rachid Saile, Hassan Taki

**Affiliations:** ^1^ Laboratory of Biology and Health, URAC 34, Faculty of Sciences Ben M’sik, Health and Biotechnology Research Center, Hassan II University of Casablanca, Casablanca, Morocco; ^2^ Mohammed VI Center for Research and Innovation, Rabat, Morocco

**Keywords:** nutritional status, carbohydrates proteins and fat intake, professional and adolescent football players, competitive period, aerobic performance, the Yo-Yo IR test

## Abstract

**Introduction:**

Nutrition plays an integral role in optimizing football players’ performance during training sessions and matches and maintaining their overall health throughout the season. This study aimed to evaluate how well the dietary practices of professional and adolescent football players in Morocco during the competitive period met international macronutrient recommendations, and to explore the relationship between their nutritional status and aerobic performance, as measured by the Yo-Yo IRL1 test.

**Methods:**

A total of 277 footballers from Morocco's professional league, “Botola-Pro”, were monitored over a seven-day (training microcycle) during the competitive period. The dietary intake was assessed through self-reported methods and 24-hour recalls. Relevant body composition was measured with bioelectrical impedance (BI), and aerobic performance was evaluated using the Yo-Yo IR test.

**Results:**

The results indicated significant variations in performance and nutritional status across different categories and age groups. The nutritional status of the players didn't match the UEFA recommendations (*p* < 0.001). We found that higher intake levels of carbohydrates and proteins were positively correlated with the total distance covered by the players (*p* < 0.001, r = 0.63, R^2^ = 0.4 for carbohydrates; *p* < 0.001, r = 0.59, R^2^ = 0.35 for proteins). Conversely, a higher proportion of energy derived from fats in the diet was negatively correlated with the distance covered (*p* < 0.001, r = −0.64, R^2^ = 0.41).

**Conclusion:**

These findings suggest that optimizing carbohydrates and protein intake while managing fat consumption is crucial for enhancing sporting performance. This information is essential for tailoring training programs and nutritional regimens based on the competition level.

## 1 Introduction

Soccer, widely regarded as the world's most popular sport, is marked by repeated bursts of high-intensity activity (1). It requires a complex set of physical qualities, skillfully combining intense phases of running, sprints, changes in direction, and jumps with moments of relative recovery (2, 3). These diverse performances, ranging from explosive accelerations to powerful movements, demand a delicate balance between anaerobic efforts and aerobic endurance (4).

Making good nutritional choices can enhance the health and performance of football players, the type, quantity, and timing of food, fluids, and supplements consumed can significantly impact players’ performance and recovery during and between matches (5). However, the rapidly evolving nature of the game, both technically and tactically, underscores the importance for medical staff and players to make informed nutritional decisions (6). These decisions are vital to supporting training loads and optimizing physical performance. A recent meta-analysis revealed that over the past two decades, from 2000 to 2019, players whether professionals, amateurs, adults, or adolescents have faced challenges in effectively meeting their nutritional needs, particularly concerning energy and carbohydrates intake (7).

Several recommendations have been implemented over time, with the most recent being the UEFA consensus (5)suggesting that one day before the match (D-1), the match day (D), and the day after the match (D + 1), carbohydrate (CHO) intake should be increased to replenish muscle glycogen reserves. It's recommended to consume 6–8 g/day/kg of body weight (BW) (5).

The proteins, composed of amino acids, play a crucial role in the repair and growth of muscle tissues solicited during aerobic efforts (8).They also contribute to the synthesis of new enzymes and molecules essential for performance. Muscle recovery after an intensive football match involves protein synthesis, where amino acids from dietary proteins are used to repair damaged muscle fibers (9). This muscular regeneration helps minimize fatigue, prevent injuries, and support long-term athletic performance (10). Moreover, in the context of football, where endurance, speed, and strength are crucial, proteins play a key role in the synthesis of enzymes and molecules necessary for energy production (11). Enzymes facilitate metabolic processes that provide the necessary energy to sustain the intensity of the game (12–14). Adequate protein intake in the diet thus contributes to optimizing the performance of football players on the field. The UEFA recommendations suggest a protein intake ranging from 1.2 to 2.2 g/day/kg of body weight (BM) for players (5), and according to recent studies conducted primarily in Europe, the USA, Australia, and Asia, most adults often meet this ratio (7). The scientific community suggests that adolescents should also have sufficient protein intake during this crucial phase to promote normal growth and development in young footballers (15).

The dietary fat plays a crucial role in training nutrition by serving as an energy source, facilitating the absorption of fat-soluble vitamins, and providing essential fatty acids. Athletes are recommended to tailor their fat intake to meet protein and carbohydrate requirements within overall energy goals (16). Additionally, adherence to community guidelines regarding minimal trans fatty acid intake and cautious consumption of saturated fats is advised (17) The UEFA consensus recommends that the percentage ratio for fat intake should fall within the range of 20%–35% of total energy intake (TEI) (5).

In sport performance, the aerobic capacity (AC), represents an essential element of physical fitness (PF) that defines the ability of players to maintain their optimal performance levels throughout a match or an intense training session (12). Many research studies have been carried out in the last few years to investigate the relationship between running distance covered, and physical performances during football games, both at the semi-elite and youth levels (18–23), and at the professional level (1, 12, 24–28). The results of these studies confirm a strong correlation between physical fitness and the running demands during matches, highlighting that total distance covered and peak speed in endurance tests are associated with both total distance and high-intensity distance covered during play. In this context, field tests are considered a more appropriate alternative to laboratory tests (19, 29), mainly due to their relevance to the sport, low cost, and their ease of implementation. Considering these aspects, it will appropriate use field tests during the competitive season frequently.

The physical fitness (PF) of football players engaged in significant competitive levels has been the subject of thorough analysis over the past decades, utilizing various testing protocols, including the Yo-Yo IR1 test developed by Bangsbo et al. (30) which involves covering the greatest possible distance while following a running pattern that includes periods of intense effort and recovery. The assessment of seasonal variability in players’ physical capacity within different European football leagues has been conducted using this protocol and similar ones (27, 31–34).

On the other hand, several scientific studies have shown that footballers, whether adults or adolescents, struggle to meet their nutritional needs, whether in terms of energy or macronutrients (7). Furthermore, adolescents face a paradox: they must not only meet the demands of sports performance but also satisfy the specific needs related to their growth phase… (significant changes, physically psychologically …). The combination of intense training, competitive demands, and physiological changes can increase adolescents’ vulnerability to phenomena such as fatigue, overtraining, and injuries. Prolonged fatigue not only can affects sports performance but also impacts negatively the overall wellbeing and growth of adolescents (26, 35). On the flip side, making optimal nutrition choices is crucial for promoting health and performance. The quality, quantity, and timing of food, fluids, and supplements consumption play a pivotal role in influencing the players’ performance and recovery time both during and between matches (5, 36).

Following this introduction, it is essential to highlight the crucial importance of the relationship between nutritional status and aerobic performance, whether during matches or training. This relationship is influenced by various factors, including the level of competition, age category, workload intensity, exertion, and other parameters. However, to date, and to our knowledge, a few studies have explored these relationships, particularly in Morocco (37), North Africa, and across the African continent. Therefore, this study aims to (I) evaluate the dietary habits and nutritional status in terms of macronutrients among Moroccan adolescent and adult football players, to determine if their diet aligns with the international recommendations of the UEFA scientific community for adults and professionals, as well as with Moroccan nutritional recommendations for pre-adolescents; (II) examine the impact of this nutritional status on aerobic performance, measured by the distance covered during the Yo-Yo IR1 test.

In this context, it is crucial to examine several questions to deepen our understanding of the interactions between nutrition and performance in Moroccan football. This study aims to address the following questions:

1. Do Moroccan football players adhere to macronutrient recommendations?
2. Do players with higher levels of aerobic performance better comply with macronutrient nutritional recommendations?
3. Is there a correlation between the level of competition and the nutritional status of players?
4. How do players’ dietary habits evolve from adolescence to adulthood, and are these changes aligned with nutritional recommendations for different levels of competition?
5. What is the impact of nutritional status on aerobic performance, as measured by the distance covered during the Yo-Yo IR1 test?

These questions seek to clarify the complex relationships between nutritional intake and sports performance, considering the specificities of Moroccan players across various levels of competition.

## 2 Material and methods

### 2.1 Study design

**Figure 1:**
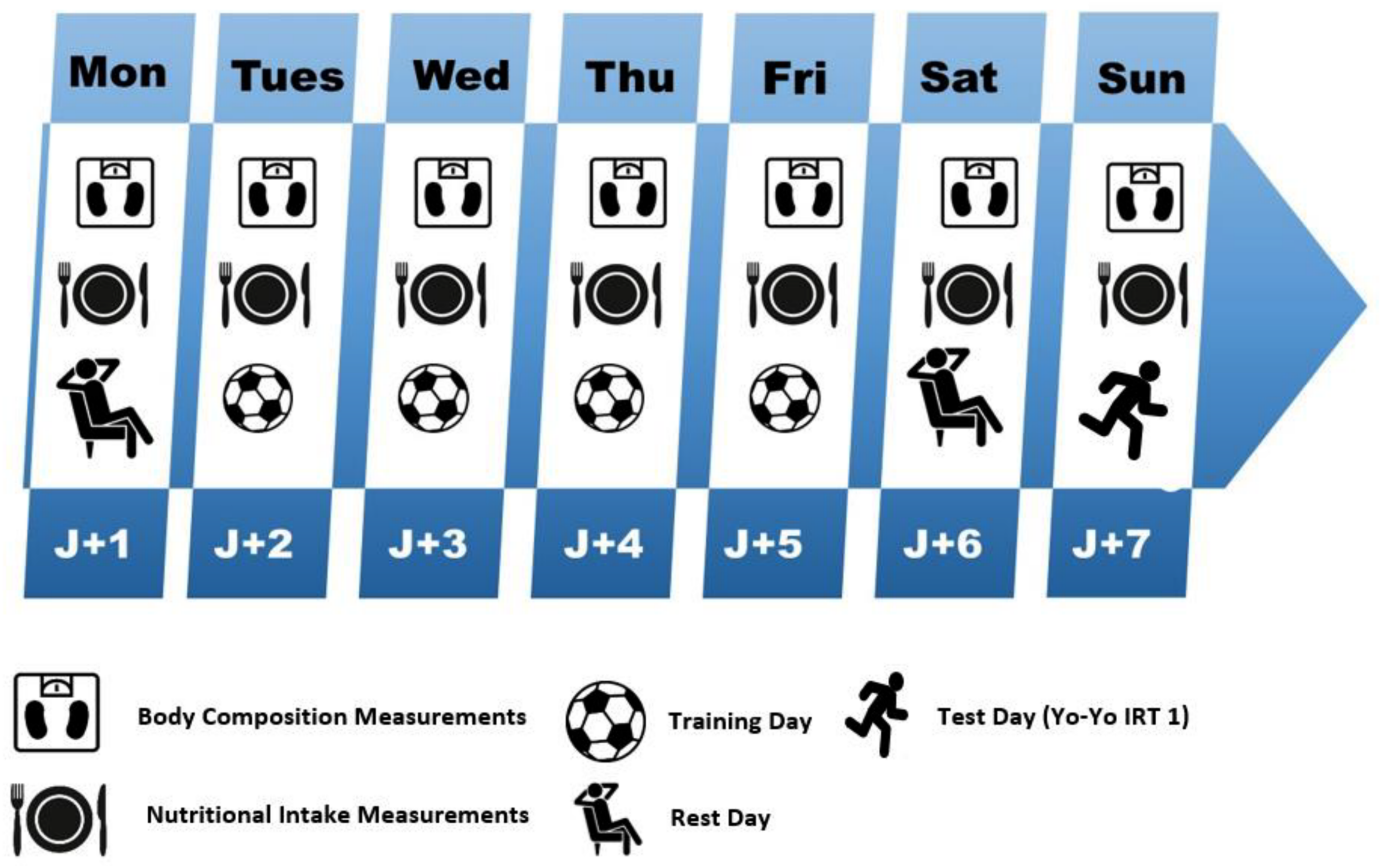
The study design.

An observational study protocol was established to assess potential relationships between macronutrient intake, energy intake, and physical performance measures expressed in distance covered following a validated field test Yo-Yo TR1 (30). The aim was to investigate the impact of nutritional status on performance in a total of 253 male soccer players in Morocco of varying ages (from 12 to 25), from academic to professional level competition. The study was conducted during the second half of the return phase (after transition phase) of the Moroccan “Botola-Pro” National league Championship competitive season 2022–2023. Each age category or group was monitored over a period of 7 consecutive days, including one microcycle training from Monday to Saturday, as illustrated in Figure 1.

Dietary survey and anthropometric measurements were conducted daily from monday to sunday at 10 a.m. before training sessions. It's noteworthy that the physical test was conducted during the Sunday session, and no other activity was carried out before or during this testing period to ensure the validity of the protocol and to avoid any other factors that could influence the results. A warm-up of 5–10 min was performed based on the qualities of each player, as well as their category and competition level.

This study has been approved by the regional ethics committee of the Ibn RORCHD University Hospital Center of Casablanca governed by Morocco's Ministry of Health (Approval No. 22/2022) and has been conducted in accordance with the ethical principles outlined in the Declaration of Helsinki. The participants have given their consent to be involved in the study and were given the option to opt out at any moment.

### 2.2 Participants

A total of 277 male Moroccan football players from 14 clubs competing in the “Botola-Pro” Moroccan championship were included in the study, with careful distribution made across age groups and competitive levels. The eligibility criteria applied to this study excluded goalkeepers (a number of 14) because the physical effort exerted by goalkeepers differs from that of other players, and our objective is to evaluate the aerobic performance of outfield players. Also excluded were the players who did not complete performance tests (a number of 4) or the dietary questionnaires (totaling 5), as well as those with injuries or illnesses during the research period (a number of 2).

In total 253 participants representing 14 football teams with players (ranging from pre-adolescence to adulthood, from academic to professional levels), were followed, analyzed, and processed till the end of the study and distributed as follows:

The *Senior Professional group level “S-PROF”* was made up of 49 players from two professional clubs that were involved in the “Botola-Pro” competition. Fifty players from two teams competing in the national elite tournament made up the senior elite group, or “S-ELIT”. Fifty players from two teams competing in the young elite division of the national elite youth championship made up the “Y-ELIT” group of young players. Finally, 55 players from three teams in the U15 and U14 categories (referred to as “U15/U14-ACDM”) who were competing in the academic championship were also included in our study. Furthermore, 49 competitors in the U13 and U12 divisions were also included.

### 2.3 Anthropometry

InBody Co., Ltd.'s InBody 120 bioelectrical impedance analyzer was used to collect anthropometric data. This analyzer produces a current of 150 μA (± 50 μA) while operating at frequencies of 20 and 100 kHz. It offers a range of complete findings, including weight, body mass index (BMI), body fat mass (BFM), body muscle mass (BMM), and Resting Metabolic Rate (RMR). The precision errors less than 2% for lean body mass (FFM), fat mass (FM), and percentage of body fat (%BF) (38, 39).

A stadiometer (Portable Stadiometer-Seca 225, Hamburg, Germany) with an accuracy of ±0.1 mm was used to measure the subjects’ height; to assure accuracy, athletes took off their shoes.

The World Health Organization (WHO) curves were used in conjunction with a particular approach to calculate BMI for participants in the Under 18 group. These globally accepted curves ensure an accurate evaluation of weight status while accounting for changes linked to growth and physiological development (40).

### 2.4 Physical performance test

To assess the participants’ physical performances, we opted for the Yo-Yo intermittent recovery test level 1 (Yo-Yo IRT 1) developed by Bangsbo et al. (30), a recognized measure in the field of aerobic endurance evaluation, this test involves a series of repeated runs with progressively reduced recovery intervals. Participants are pushed to their limits in terms of speed and endurance, providing a reliable assessment of their aerobic capacities. The Yo-Yo IRT 1 test is often favored for its specificity to the demands of football, challenging players’ ability to sustain high-intensity efforts throughout the test. the distance traveled is expressed in meters (m) and the results obtained through the Yo-Yo test will contribute to our in-depth understanding of the participants’ physical performance profiles within the context of our study.

### 2.5 Nutritional status measurement

A rigorous protocol was implemented for the collection of nutritional data, aiming to comprehensively assess the intake of macronutrients, including carbohydrates, proteins, and fats, as well as dietary fiber and cholesterol among the examined players. For each category, the 24-hour dietary recall method was systematically applied over a period of seven consecutive days (Figure 1). This approach involved the use of standard household units, such as spoons, glasses, and containers, to enable a precise estimation of the quantities consumed (41, 42). Additionally, the Remote Food Photographic Method (RFPM) was integrated into the protocol as a complementary method to meticulously estimate caloric intake. This methodological decision aligns with recommendations from scientific studies, particularly the work of Martin et al. (43). The utilization of RFPM added an extra dimension to our assessment of energy intake, providing a detailed visual perspective on the dietary habits of players throughout the study duration.

### 2.6 Dietary analysis

A careful analysis of dietary intake was conducted using the Nutrilog 3.30 software, a tool based on the 2020 Ciqual nutritional database (44, 45). This software provides an advanced platform that accurately determines the levels of macronutrients such as carbohydrates, proteins, and fats, as well as micronutrients, fibers, and various other food components. Leveraging the updated Ciqual database, Nutrilog software facilitated a thorough analysis of participants’ nutritional intake, thereby offering a comprehensive insight into the composition of their dietary patterns during the study period.

### 2.7 Statistical analysis

The statistical analysis of the data collected was conducted using SPSS version 27 (IBM, SPSS Statistics, Version 27, Chicago IL), as the primary analytical tool. The mean (mean) was employed to represent the central tendency of the data and the Standard deviation (SD) was used to assess the dispersion or variability around the mean.

To assess the normality of variables, the Kolmogorov-Smirnov test was applied. The *T*-test was used form comparisons of the means between macronutrient intake and UEFA recommended values for professional and elite and young groups, as well as between means of Moroccan recommendations for academic levels (U15/U14 and U13/U12). And a one-way analysis of variance (ANOVA) with post-hoc testing and the Tukey test to identify the level of macronutrient consumption that could enhance the athletic performance of the studied football players. For carbohydrates, players were categorized into four groups based on the quantity consumed: less than 4 g/kg body weight, between 4 and 5 g/kg body weight, between 5 and 6 g/kg body weight, and over 6 g/kg body weight. For protein intake, participants were classified into three groups: less than 1.6 g/kg body weight, between 1.6 and 2.2 g/kg body weight, and over 2.2 g/kg body weight. To explore the impact of fat intake on sports performance, participants were grouped into four categories based on the quantity of fats consumed (between 20% and 25%, between 25% and 30%, between 30% and 35%, and over 35%).

The Pearson correlation test was applied to examine the relationships between various performance variables and dietary intake. Statistical significance was assessed with a threshold of *p* < 0.05, indicating a level of significance. Additionally, a 95% confidence interval (CI) was set to enhance result precision. These analytical procedures, along with the defined significance criteria, were rigorously adhered to ensure the validity and reliability of the conclusions drawn from this study.

## 3 Results

### 3.1 Anthropometric characteristic's

Table 1 presents a descriptive analysis of the anthropometric characteristics of the 253 players studied, distributed across different age categories. Taking into account the level of competition, body mass index, basal metabolism, and body fat percentage, this table shows a trend of increasing values according to age, except for the body fat percentage, which decreases with age and increases with the level of competition with averages of 14.3% (±1.2) to 10.2% (±2.3).

**Table 1:** Descriptive analysis of the sample characteristics (participants players 253).

Variables	S-PROF		S-ELIT		Y-ELIT		U15/U14 ACDM		U13/U12 ACDM	
	Mean	±SD	Mean	±SD	Mean	±SD	Mean	±SD	Mean	±SD
Age (years)	25.2	3.4	21.5	2.4	16.0	0.4	14.8	0.4	12.8	0.4
Height (cm)	179.8	6.4	177.9	4.4	172.5	4.1	165.1	5.8	155.1	6.2
Weight (kg)	72.6	6.0	70.6	5.1	63.1	4.9	55.3	4.6	47.2	7.1
BMI	22.4	1.2	22.3	1.2	21.2	1.2	20.3	1.2	19.5	2.3
BFM (%)	10.2	2.3	12.1	1.3	12.5	1.2	13.4	1.2	14.3	1.2
BM (Kcal)	1794	109	1782	84	1689	81	1552	84	1404	119

BMI, body mass index; BFM, body fat mass; BM, Basal metabolic; SD, standard deviation; *p*-value at 0,05. Senior professional “S-PROF” senior elite group level “S-ELIT”, Young elite group level “Y-ELIT”, U15 and U14 categories academic level “U15/14-ACDM”. U13 and U12 categories academic level “U13/12-ACDM”.

### 3.2 Performance

The performance results for Yo-Yo IRT measurements are presented in Table 2, indicating means (m) with standard deviations (±SD), detailed for each category. The normality of all variables was tested using the Kolmogorov-Smirnov test, which displayed a *p*-value greater than 0.05. To determine significant differences in performance, especially between professional and elite levels, as well as between academic levels, the ANOVA test was utilized with post-hoc testing and Tukey's test. The results revealed statistically significant variations (F = 175.86) among the means of the S-PROF, S-ELIT, Y-ELIT, U15/14-ACDM, and U13/12-ACDM categories (*p* < 0.001). Professional players (S-PROF) exhibited superior performances, covering a distance of 2,451.5 m ± 162.9, while S-ELIT, U15/14-ACDM, and U13/12-ACDM achieved distances of 2,200.8 m ± 380.8; 2,101.6 m ± 331.2; 1,677.8 m ± 209.0; and 1,175.1 m ± 174.5, respectively.

**Table 2:** The physical test (Yo-Yo IR1) and significance differences by ANOVA statistical test for All groups of football players.

(I) categories (*N* = 253)	Mean (m)	±SD	(J) Group of categories	Mean difference (I-J)	Sig.	95% Confidence interval	
						Lower bound	Upper bound
S-PROF	2451.5	162.9	S-ELIT	250.7306^a^	<0.001	103.688	397.773
			Y-ELIT	349.9306^a^	<0.001	202.888	496.973
			U15/14-ACDM	773.7124^a^	<0.001	630.016	917.409
			U13/12-ACDM	1276.4286^a^	<0.001	1128.645	1424.212
S-ELIT	2200.8	380.8	S-PROF	−250.7306^a^	<0.001	−397.773	−103.688
			Y-ELIT	99.2000	0.340	−47.098	245.498
			U15/14-ACDM	522.9818^a^	<0.001	380.047	665.916
			U13/12-ACDM	1025.6980^a^	<0.001	878.655	1172.741
Y-ELIT	2101.6	331.2	S-PROF	−349.9306^a^	<0.001	−496.973	−202.888
			S-ELIT	−99.2000	0.340	−245.498	47.098
			U15/14-ACDM	423.7818^a^	<0.001	280.847	566.716
			U13/12-ACDM	926.4980^a^	<0.001	779.455	1073.541
U15/14-ACDM	1677.8	209.0	S-PROF	−773.7124^a^	<0.001	−917.409	−630.016
			S-ELIT	−522.9818^a^	<0.001	−665.916	−380.047
			Y-ELIT	−423.7818^a^	<0.001	−566.716	−280.847
			U13/12-ACDM	502.7161^a^	<0.001	359.020	646.413
U13/12-ACDM	1175.1	174.5	S-PROF	−1276.4286^a^	<0.001	−1424.212	−1128.645
			S-ELIT	−1025.6980^a^	<0.001	−1172.741	−878.655
			Y-ELIT	−926.4980^a^	<0.001	−1073.541	−779.455
			U15/14-ACDM	−502.7161^a^	<0.001	−646.413	−359.020

Senior professional “S-PROF” senior elite group level “S-ELIT”, Young elite group level “Y-ELIT”, U15 and U14 categories academic level “U15/14-ACDM”. U13 and U12 categories academic level “U13/12-ACDM”.

^a^ The mean difference is significant at the 0.01 level (ANOVA test).

Significant differences were observed between the S-ELIT, U15/14-ACDM, and U13/12-ACDM categories (*p* < 0.001), as well as between the Y-ELIT, U15/14-ACDM, and U13/12-ACDM categories (*p* < 0.001). However, no difference was found between the S-ELIT and Y-ELIT categories, with a *p*-value of 0.34.

### 3.3 Dietary status

**Figure 2:**
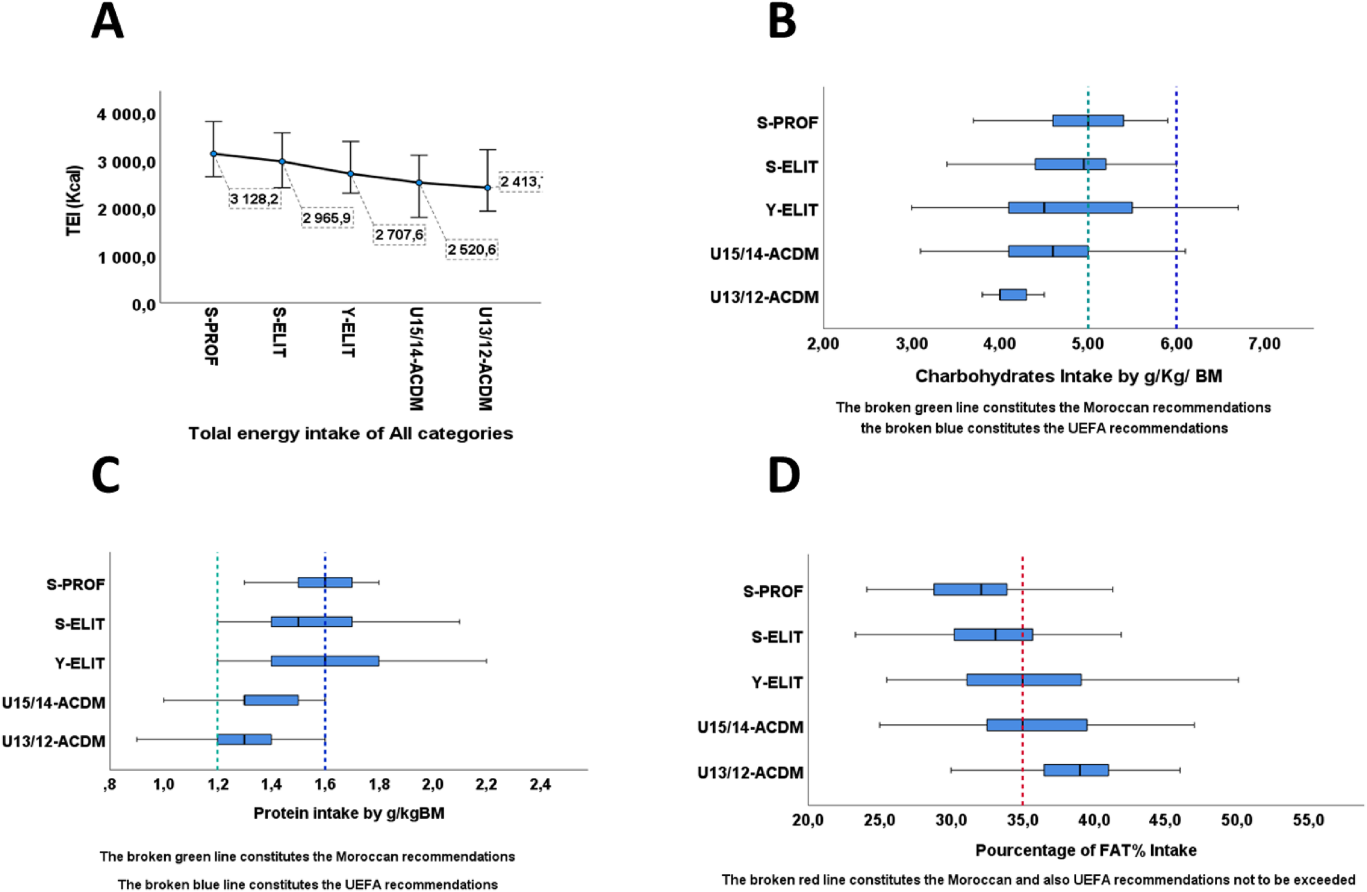
The distribution of macronutrients consumed by players from different categories in accordance with the recommendations. **(A)** The distribution of Total Energy intake of all categories. **(B)** The distribution of carbohydrates intake by g/kg/BM of all categories. **(C)** The distribution of protein intake by g/kg/MB of all categories. **(D)** The distribution of Fat intake of all categories.

Table 3 presents data on the athletes (*N* = 253 participants) macronutrient consumption as means with standard deviations (±SD). The total energy intake (TEI) ranges from 2,414 to 3,128 kcal (Figure 2A, Table 3). For the U15/14 and U13/12 categories (academic level), the energy intake aligns with Moroccan recommendations, with values of 2,521 ± 260 and 2,414 ± 300 Kcal, respectively. However, it significantly deviates for competitive-level footballers according to UEFA standards, with a *p* < 0.001. This trend is also evident in carbohydrate (CHO) consumption in g/kg of body mass, showing values of 4.5 g/kg BM for U15/14 and 4.2 g/kg BM for U13/12.

**Table 3:** Macronutrient intake compared to recommendations of all football players groups.

Total (*N* = 253)	Mean of variables and recommendations (5)			*p-value*
S-PROF (*N* = 50)	Mean	±SD	DRIs	
TEI (Kcal)	3128	295	3400–4300	<0.01
CHO%	53	4	———–	——
CHO (g/kg BM)	5.0	0.5	6–8	<0.01
PRO (g/kg BM)	1.7	0.2	1.6–2.2	0.07
PRO%	15	2	———–	——
FAT (g/kg BM)	1.5	0.2	———–	——
FAT%	31.8	4.0	20–35	<0.01
Fiber (g)	27.1	1.7	20–40	<0.01
Choles (mg)	324	84	< 300 mg	<0.01
S-ELIT (*N* = 50)				
TEI (Kcal)	2966	242	3400–4300	<0.001
CHO%	52	4	———–	——
CHO (g/kg BM)	4.8	0.6	6–8	<0.01
PRO (g/kg BM)	1.6	0.2	1.6–2.2	0.63
PRO%	15	2	———–	——
FAT (g/kg BM)	1.6	0.2	———–	——
FAT%	33.2	4.5	20–35	<0.01
Fiber (g)	24.8	2.8	20–40	<0.01
Choles (mg)	404	66	< 300 mg	<0.01
Y-ELIT (*N* = 50)				
TEI (Kcal)	2708	233	3400–4300	<0.001
CHO%	50	6	———–	——
CHO (g/kg BM)	4.7	0.9	6–8	<0.01
PRO (g/kg BM)	1.6	0.2	1.6–2.2	0.17
PRO%	15	2	———–	——
FAT (g/kg BM)	1.7	0.3	———–	——
FAT%	35.2	5.8	20–35	<0.01
Fiber (g)	25.4	2.8	20–40	<0.01
Choles (mg)	385	63	< 300 mg	<0.01
U15/U14 ACDM (*N* = 50)				
TEI (Kcal)	2521	260	2500–3000	<0.05
CHO%	50	6	———–	——
CHO (g/kg BM)	4.5	0.7	5	<0.01
PRO (g/kg BM)	1.4	0.2	1.2	
PRO%	13	2	———–	——
FAT (g/kg BM)	1.8	0.3	———–	——
FAT%	36.3	6.4	20–35	<0.01
Fiber (g)	21.6	3.2	20–40	<0.01
Choles (mg)	415	63	< 300 mg	<0.01
U13/U12 ACDM (*N* = 50)				
TEI (Kcal)	2414	300	2500–3000	<0.05
CHO%	47	6	———–	<0.01
CHO (g/kg BM)	4.2	0.7	5	<0.001
PRO (g/kg BM)	1.4	0.2	1.2	
PRO%	13	1	———–	——
FAT (g/kg BM)	2.1	0.3	———–	——
FAT%	39.2	6.3	20–35	<0.01
Fiber (g)	19.2	3.6	20–40	<0.01
Choles (mg)	371	73	< 300 mg	<0.01

TEI, Total energy intake; CHO, Carbohydrates; PRO, Proteins. FAT% ration of fat energy; Choles: Cholesterols. Senior professional “S-PROF” senior elite group level “S-ELIT”, Young elite group level “Y-ELIT”, U15 and U14 categories academic level “U15/14-ACDM”. U13 and U12 categories academic level “U13/12-ACDM” DRIs: Dietary Recommendation Intake value.

Moreover, the S-PROF (3,128 ± 295 Kcal), S-ELIT (2,966 ± 242 Kcal), and Y-ELIT (2,708 ± 233 Kcal) groups exhibit slightly lower levels compared to UEFA recommendations for professional and elite players, with significant differences of *p* < 0.001 and *p* < 0.01 concerning the recommended values for energy intake. This trend is also reflected in carbohydrate intake (CHO) in g/kg of body weight, with quantities of 5.0 g/kg, 4.8 g/kg, and 4.7 g/kg of body mass (BM) respectively for the S-PROF, S-ELIT, and Y-ELIT groups, displaying statistical significance of *p* < 0.01 (Figure 2B, Table 3). Also, the percentage of carbohydrates (CHO%) varies from 53%, 52%, and 50%.

However, the protein intake (PRO) in the S-PROF, S-ELIT, and Y-ELIT groups aligns with UEFA recommendations, with values of 1.7 g/kg, 1.6 g/kg, and 1.6 g/kg of body mass (BM) with a value of 15% of total energy consumption. As for players in the U15/14-ACDM and U13/12-ACDM categories, they adhere to Moroccan recommendations but are slightly below the guidelines for competitive athletes, with a value of 1.4 g/kg for each category (Figure 2C, Table 3).

Moreover, an increased consumption of fats (FAT%) is clearly evident: Only the professionals (27%) do not exceed the limits stated as recommendations; the other categories often exceed the recommended levels, presenting values of 35.2%, 36.3%, and 39.2%, respectively, for the Y-ELIT, U15/14 ACDM, and U13/12ACDM categories (Figure 2D, Table 3). Simultaneously, an excess of cholesterol levels (Choles) has been observed, with values ranging from 324 to 415 mg. The young players exhibit higher values compared to professionals (324 mg ± 63) overall, in Y-ELIT, U15/14ACDM, and U13/12ACDM categories: 385 mg ± 63, 415 mg ± 63, and 371 mg ± 73, respectively.

Regarding fiber intake, players have quantities ranging from 19.2 to 27.1 g. Young players exhibit lower levels of fiber compared (*p < 0,01)* to those of professionals and elite-level players.

## 4 Discussion

The main objective of this study was twofold: firstly, to assess and describe the dietary habits as well as the nutritional status in terms of macronutrients among Moroccan football players, taking into account their level of competition. This analysis explored the evolution of these aspects from adolescence to adulthood, including the transition to the professional level, in order to assess whether these dietary patterns align with international recommendations (5, 46).

Secondly, to establish whether there are correlations between dietary choices and physical performance in Moroccan’s football players at different stages of development.

### 4.1 The players show deficiencies in meeting the recommended macronutrient intake

Like all other findings on an international scale (6, 47, 48), Moroccan players exhibit energy and carbohydrate intake levels below the established recommendations, a recurring observation across all player categories. This deficit may be attributed to specific dietary habits, which seem not to fully meet the nutritional requirements of high-level sports. In contrast, protein intake generally adheres to the recommendations, particularly among adults, indicating a better awareness of the importance of protein in the athletic diet. However, fat intake consistently exceeds recommendations across all categories, likely due to the consumption of fast food that is high in fat.

This situation, also observed in previous studies (7), is very common among high-level athletes, where nutritional imbalances are often noted, especially in competition. This raises concerns about the nutritional balance of the players, suggesting that a reevaluation of dietary choices is necessary to align their diet with international guidelines and optimize their sports performance.

The analysis of data on energy intake and macronutrient consumption among the studied football players demonstrate notable variations. The total energy intake (TEI) generally ranges between 2,414 and 3,128 kcal with significant differences observed among categories based on the level of competition. (Table 3). The S-PROF (3,128 ± 295 Kcal), S-ELIT (2,966 ± 242 Kcal), and Y-ELIT (2,708 ± 233 Kcal) groups show slightly lower energy intake levels compared to UEFA (5) (Union of European Football Associations) consensus sports nutrition recommendations, a significant divergence was found with a *p*-value 0.01, for professional and elite players. While the academic-level categories U15/14 ACDM (2,521 ± 260 kcal) and U13/12 ACDM 2,414 ± 300 Kcal diverge considerably from UEFA standards for competitive-level players *p*-value 0.001. (Table 3). Although they align more closely with Moroccan recommendations, there are still disparities to consider.

The observed levels of energy intake are comparable to those reported in other countries among football players. Similar studies conducted with senior football players have reported estimated energy intakes ranging from 2,164 ± 498 kcal to 3,442 ± 158 kcal (7, 49, 50). A study that appears more closely aligned with our results is the one concerning the professional players conducted in Japan by Ebine et al. (51) (Japanese Professional Players aged 22 ± 2 years, 69.8 ± 4.7 kg) with an energy intake of 3,113 ± 581 for a period of 7 consecutive days. For elite players, the study conducted by Raizel et al. in 2017 (52) for a sample of 20.7 ± 2.0 years showed an TEI intake of 2,924 ± 460 kcal, and for players in the Dutch Eredivisie, the study by Bettonviel et al. (11); (Dutch Eredivisie Professional Players, for 20 players, 20 ± 4 years, 73 ± 8 kg, in 4 days in-season) reported a value at 2,988 ± 583 kcal. As for young players, the research by Galanti et al. (53), in 2015 for a population of 15–16 years old shows an energy intake of 2,844 kcal. Regarding the academic level of pre-adolescents (U13 and U12), the study by Hannon et al. (54); conducted in 2020 for players in the U12/13 category, 12 ± 0 years old, 43.0 ± 4.8 kg, indicates an intake of 2,659 kcal ± 187. All these investigations suggest that energy intakes are below the recommended levels, emphasizing the players’ struggle to meet their energy needs adequately.

On the other hand, disparities in Total Energy Intake (TEI) among the groups likely result from a combination of differences in anthropometric profiles, particularly age, basal metabolic rate (RMR), and physical load among the teams. In terms of performance, it's relevant to note that professional players, as illustrated in the figure Figure 2A, have also covered the greatest distance compared to other categories, while U13/12 players recorded the smallest distance.

**Figure 3:**
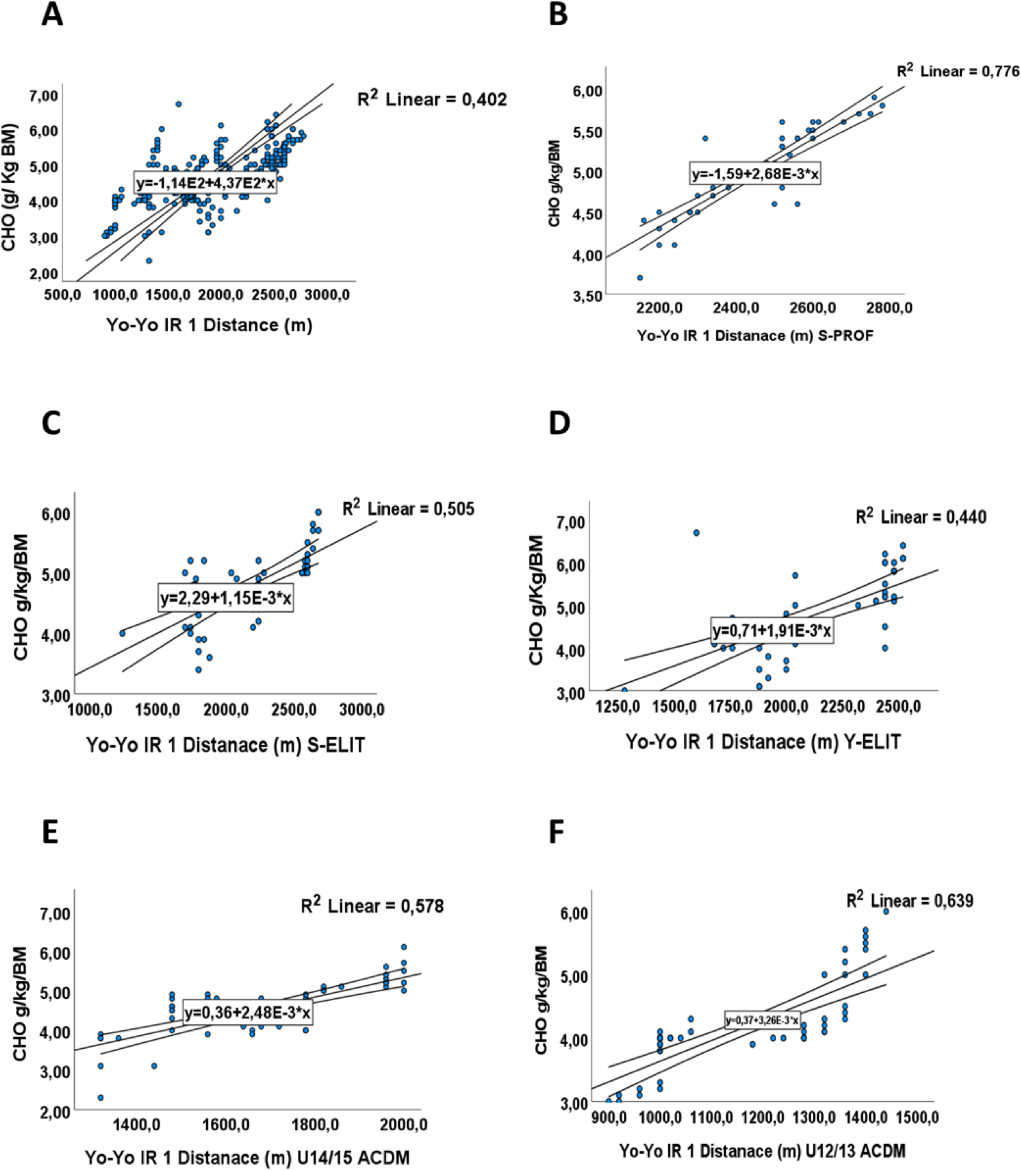
The relationship between mean distance (m) in Yo-Yo IR test and Carbohydrate's intake in g/kg/BM (CHO). **(A)** The relationship between mean distance (m) and Carbohydrate's intake in g/kg/BM (CHO) for all groups. **(B)** The relationship between mean distance covered (m) and Carbohydrate's intake in g/kg/BM (CHO) for S-PROF (Senior Professionel players). **(C)** The relationship between mean distance covered (m) and Carbohydrate's intake in g/kg/BM (CHO) for S-ELIT (Senior Elit players). **(D)** The relationship between mean distance covered (m) and Carbohydrate's intake in g/kg/BM (CHO) for Y-ELIT (Young Elit players). **(E)** The relationship between mean distance covered (m) and Carbohydrate's intake in g/kg/BM (CHO) for 14/15 ACDM (Academic players). **(F)** The relationship between mean distance covered (m) and Carbohydrate's intake in g/kg/BM (CHO) for 12/13 ACDM (Academic players).

Furthermore, following to the Pearson test, a positive correlation links was detected between TEI and age (*p* = 0.001 and r^2^ = 0.48) Figure 3D, Table 4. Also, between the TEI and RMR (*p* = 0.001 and r^2^ = 0.69) Figure 3E, Table 4, as well as between TEI and the distance covered during the field test (*p* = 0.001 and r^2^ = 0.38) Figure 3C, Table 4. Consequently, it would be advisable to consider age, resting metabolic rate (RMR), and the level of competition when developing age-specific training programs and diets to achieve adequate energy intake and meet the physical and physiological requirements of this activity.

**Table 4:** The correlation between distance covered and age, RMR, TEI and Macronutrients intake of all groups among football players.

Variables and Pearson correlation		Distance covered (m)	Age (years)	RMR	TEI	CHO (g/kg BM)	PRO (g/kg BM)	FAT%
Distance covered (m)	Pearson correlation							
	Sig. (2-tailed)							
Age (years)	Pearson correlation	0.717^a^						
	Sig. (2-tailed)	<0.001						
RMR	Pearson correlation	0.640^a^	0.692^a^					
	Sig. (2-tailed)	<0.001	<0.001					
TEI	Pearson correlation	0.620^a^	0.694^a^	0.831^a^				
	Sig. (2-tailed)	<0.001	<0.001	<0.001				
CHO (g/kg BM)	Pearson correlation	0.634^a^	0.299^a^	0.179^a^	0.442^a^			
	Sig. (2-tailed)	<0.001	<0.001	<0.004	<0.001			
PRO (g/kg BM)	Pearson correlation	0.595^a^	0.407^a^	0.255^a^	0.399^a^	0.617^a^		
	Sig. (2-tailed)	<0.001	<0.001	<0.001	<0.001	<0.001		
FAT%	Pearson correlation	−0.646^a^	−0.391^a^	−0.215^a^	0.375^a^	−0.858^a^	−0.719^a^	
	Sig. (2-tailed)	<0.001	<0.001	<0.001	<0.001	<0.001	<0.001	

TEI, total energy intake; CHO, carbohydrates; PRO, proteins. FAT% ration of fat energy; Choles, cholesterols.

^a^ Correlation is significant at the 0.01 level (2-tailed).

### 4.2 Performance was significantly correlated with macronutrient intake levels, particularly carbohydrates, across the studied groups

#### 4.2.1 CHO and performance

The distance covered during the performance test and the amount of carbohydrates consumed (CHO) showed a positive correlation, with a *p*-value of 0.001 (Table 4). This connection is visually depicted in Figure 3A, illustrating a linear correlation with a coefficient of determination of R^2^ = 0.40. Further insights from the ANOVA test revealed that players who consumed more than 6 g of carbohydrates per day achieved the better performance (*p*-value 0.001, F = 46.82) compared to the other groups. These results confirm that an increased consumption of carbohydrates is associated with enhanced performance in terms of the distance covered.

These findings were confirmed by a linear analysis for each category, aiming to determine the extent to which carbohydrate intake affects performance. They illustrate a linear correlation with coefficient of determination values of R2 = 0.77, R2 = 0.505, R2 = 0.44, R2 = 0.578, and R2 = 0.4639 for players S-PROF, S-ELIT, Y-ELIT, U15/14-ACDM, and U13/12-ACDM respectively in Figures 3A–F. Macronutrients in particular carbohydrates (CHO), play a vital role by providing the necessary energy, promoting recovery, and contributing to the overall wellness protection of players (42), the carbohydrates, the main source of energy for the body, are broken down into glucose during digestion, and then converted into adenosine triphosphate (ATP) through glycolysis, providing the necessary energy for aerobic activities (11). In relation to the physical demands of football, And it's known that the anaerobic system provides rapid but limited energy (10), the aerobic endurance comes into play during longer periods of game and recovery between actions. The ability to maintain an effective balance between these two energy systems is crucial for ensuring optimal performance throughout the match (1).

Our results, as previously mentioned, demonstrate the players show deficiencies in meeting the recommended of carbohydrate values, a finding also observed in similar studies conducted with senior football players have reported varied estimated carbohydrate (CHO) intakes. A study closely aligned with our results was conducted by Raizel et al. in 2017 (52) for Spanish senior players, reporting a CHO intake of 5.4 ± 1.9, and by Hassapidou (55) for the senior players with an age of 24.8 (5.5) and a CHO intake of 5.3 ± 1.9. Książek et al. in 2020 (56) also reported an average consumption of 5.1 g CHO/kg BM (body mass) in professional Polish football players. For adolescents, Naughton et al. in 2016 (57) studied adolescents players with an average age of 14.4 ± 0.5 and a CHO intake of 4.7 ± 1.4. All these investigations suggest that CHO intakes are below the recommended levels, emphasizing the players’ struggle to meet their energy needs adequately. The low consumption of carbohydrates (CHO) generally corresponds to a low overall energy intake. As discussed earlier, the literature also confirms improvements in soccer performance, both at the technical level (58, 59), and in physical aspects, due to the consumption of a carbohydrate-rich diet in CHO. To ensure adequate energy intake, it is highly recommended to adopt improved nutritional periodization (60)

All these studies suggest that CHO intakes are below the recommended levels, underscoring the challenges players face in adequately fulfilling their energy requirements. A low total calorie intake is typically correlated with low carbohydrate consumption (CHO). A carbohydrate-rich diet (CHO), as previously mentioned, has been linked to increases in soccer performance, both technically (58), physically (59), and at the technical level. It is strongly advised to use enhanced nutritional periodization in order to guarantee appropriate energy intake (60).

However, CHO play a crucial role in optimizing football players’ performance by providing a rapid and effective source of energy. During a match or intensive training, simple carbohydrates, such as those found in energy drinks and fruits, are quickly converted into glucose, offering immediate energy to the muscles and brain, this rapid availability of glucose helps maintain a high level of performance and prevents premature fatigue (61). Additionally, complex carbohydrates, such as those found in pasta and rice, are essential for glycogen storage in the muscles and liver (62). An adequate glycogen store allows players to sustain prolonged efforts and improve their endurance. After exertion, consuming sufficient sugars promotes quick recovery by replenishing depleted glycogen reserves, which is crucial for effectively preparing for the next training session or competition (5). Furthermore, adequate glucose intake supports concentration, coordination, and quick decision-making, all essential aspects of high-level play (6).

### 4.3 Protein’s intake and performance

**Figure 4:**
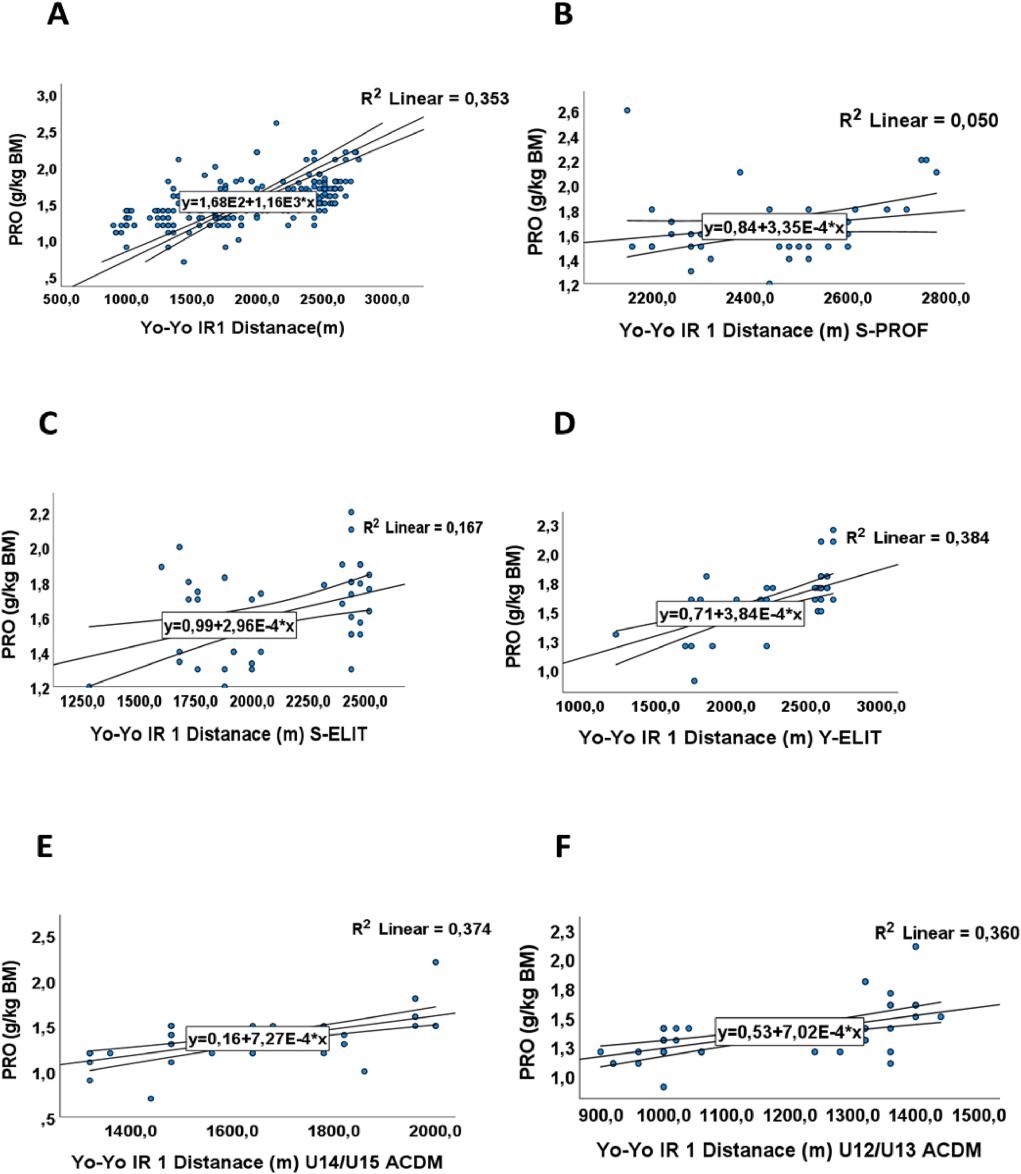
The relationship between mean distance (m) in Yo-Yo IR test and proteins intake in g/kg/BM (PRO). **(A)** The relationship between mean distance (m) and proteins intake in g/kg/BM for all groups. **(B)** The relationship between mean distance covered (m) and proteins intake in g/kg/BM for S-PROF (Senior Professionel players). **(C)** The relationship between mean distance covered (m) and proteins intake in g/kg/BM for S-ELIT (Senior Elit players). **(D)** The relationship between mean distance covered (m) and proteins intake in g/kg/BM for Y-ELIT (Young Elit players). **(E)** The relationship between mean distance covered (m) and proteins intake in g/kg/BM for U14/15 ACDM (Academic players). **(F)** The relationship between mean distance covered (m) and proteins intake in g/kg/BM for U12/13 ACDM (Academic players).

The Pearson correlation test revealed a significant positive association between protein intake levels and performance, with a correlation coefficient R^2^ of 0.64 Figure 4A and (*p*-value 0.001(Table 4). These results were validated through linear analysis within each category, aiming to assess the impact of protein intake on performance. They demonstrate a linear relationship, as evidenced by coefficient of determination values of R2 = 0.05, R2 = 0.167, R2 = 0.384, R2 = 0.374, and R2 = 0.360 for players S-PROF, S-ELIT, Y-ELIT, U15/14-ACDM, and U13/12-ACDM respectively in Figures 4B–F). This indicates that despite the importance of proteins for the energy process, this nutrient has a greater impact on the performance of young people and adolescents compared to adults, due to the growth and development phase of adolescents.

Nevertheless, when examining protein intake (PRO) in the S-PROF, S-ELIT, and Y-ELIT groups, it is noteworthy that their consumption aligns closely with the guidelines set by UEFA (Table 3). Specifically, protein intake values for these groups stand at 1.7 g/kg, 1.6 g/kg, and 1.6 g/kg of body mass (BM) respectively, constituting 15% of their total caloric intake. On the other hand, players falling under the U15/14-ACDM and U13/12-ACDM categories adhere to Moroccan dietary recommendations, albeit slightly below the suggested levels for competitive athletes. In these categories, protein intake has reported at 1.4 grams per kilogram of body mass, indicating a marginally lower adherence to the guidelines established for athletes engaged in competitive sports. A meta-analysis revealed that protein intake fluctuated within the range of 1.8–2.0 g/kg/day among junior athletes and 1.8–1.9 g/kg/day in their senior counterparts (7).

On the other hand, These results are consistent with the consensus within the scientific community, indicating that maintaining sufficient protein intake could play a pivotal role in enhancing athletes’ physical performance (63). Essentially, this recommendation is underscored by the fact that adult athletes are often advised to increase their protein consumption compared to inactive individuals (47).

However, Proteins are essential for football players due to their crucial role in muscle repair and growth for young peoples. During intense training and matches, muscles undergo micro-tears that require repair to strengthen and rebuild muscle tissue. Proteins provide the necessary amino acids for this recovery process, thereby promoting muscle adaptation and performance enhancement (15). Additionally, adequate protein intake helps maintain optimal muscle mass, which is vital for strength, speed, and agility on the field. After exertion, proteins aid in reducing muscle soreness and accelerating recovery, allowing players to return to peak performance more quickly. By incorporating sufficient protein into their diet, footballers can maximize their physical potential and improve their ability to meet the high demands of the sport (63).

### 4.4 Fat intake and performance

The acceptable macronutrient distribution range for fats is 20 to 35%, the soccer players are recommended to consume less than 30% of their total energy needs from fats (5). Our findings indicate that only the professionals (27%) do not exceed the limits stated as recommendations (Table 3), the other categories often surpass the recommended levels, so that the U13/12 ACDM>U15/14 ACDM>Y-ELI>S-ELIT T>S-PROF. Concurrently, there has been an excess of cholesterol (Choles) measured, with values varying from 324 to 415 mg. The young players show a higher value overall [Y-ELIT (385 mg ± 63), U15/14 ACDM (415 mg ± 63), and U13/12 ACDM (371 mg ± 73)] than the S-PROF (324 mg ± 63).

**Figure 5:**
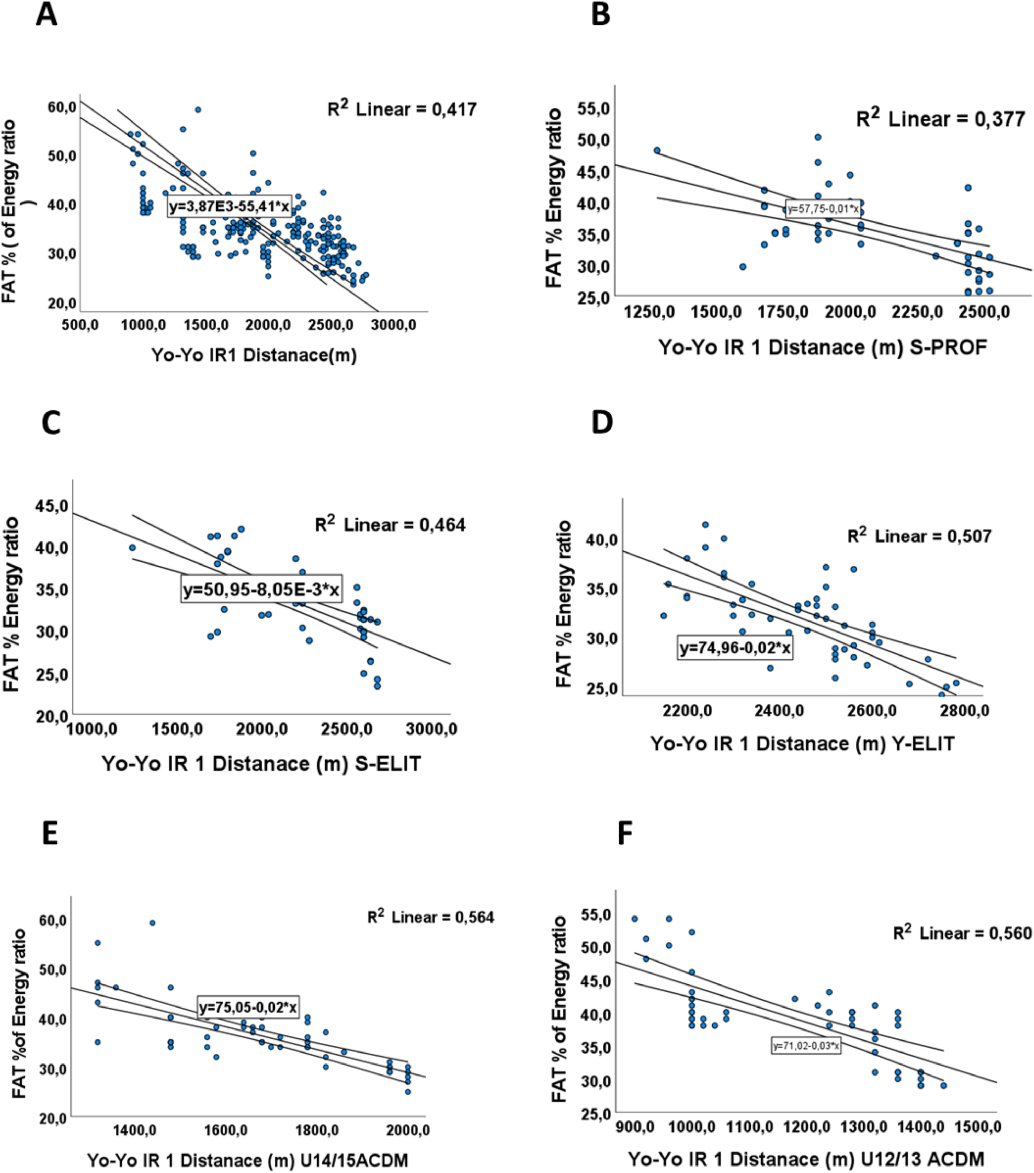
The relationship between mean distance (m) in Yo-Yo IR test and Fat intake in (FAT %). **(A)** The relationship between mean distance (m) and Fat intake in (FAT %) for all groups. **(B)** The relationship between mean distance covered (m) and Fat intake in (FAT %) for S-PROF (Senior Professionel players). **(C)** The relationship between mean distance covered (m) and Fat intake in (FAT %) for S-ELIT (Senior Elit players). **(D)** The relationship between mean distance covered (m) and Fat intake in (FAT %) for Y-ELIT (Young Elit players). **(E)** The relationship between mean distance covered (m) and Fat intake in (FAT %) for U14/15 ACDM (Academic players). **(F)** The relationship between mean distance covered (m) and Fat intake in (FAT %) for U12/13 ACDM (Academic players).

A negative correlation was observed between the amount of fat consumed and distance covered with a *p*-value = 0,001, (r = −0.64) Table 4, and R^2^ = −0.41 Figure 5A, the Players’ performance was affected by higher fat consumption, especially if it was more than 35%, according to the ANOVA test (with post-hoc testing and the Tukey test) that showed a (*p* < 0.001, F = 43.95). Our results aligned with other research on elite or professional athletes (7).

These results were validated through linear analysis within each category, aiming to assess the impact of fat intake on performance. They demonstrate a linear relationship, as evidenced by coefficient of determination values of R2 = −0.377, R2 = −0.464, R2 = −0.507, R2 = −0.564, and R2 = −0.560 for players S-PROF, S-ELIT, Y-ELIT, U15/14-ACDM, and U13/12-ACDM respectively in Figures 5B–F.

The negative values of the coefficient of determination (R^2^) observed for fat intake and performance across all player categories suggest an inverse relationship between fat intake and performance. These findings imply that higher fat intake might be associated with lower performance levels among football players in each category. It noted that fats play a crucial role in footballers’ nutrition by providing a concentrated and sustained source of energy. While carbohydrates are the primary energy source for high-intensity efforts, lipids become an important energy source during periods of low intensity and prolonged activities. Essential fatty acids, found in vegetable oils, nuts, and fatty fish, are also crucial for cell health and hormone production. Furthermore, lipids help protect internal organs and regulate body temperature, which is important for maintaining optimal performance. Balanced fat consumption enhances endurance and supports effective recovery by providing a sustained energy reserve. However, it is important to avoid unhealthy fats, such as trans fats and excessive saturated fats found in processed foods and fried items, as they can negatively impact cardiovascular health and overall athletic performance.

As a conclusion the nutritional deficiencies in macronutrients observed among professional and adult players can be attributed to several complex factors. Firstly, limited access to a varied and balanced diet, particularly in terms of fiber from fruits and vegetables, as well as essential oils such as omega-3 and omega-6, remains a significant challenge. Additionally, the lack or absence of adequate meal planning around training sessions and matches exacerbates this issue. The absence of personalized nutritional monitoring, combined with insufficient management of dietary intake and hydration, also contributes to diets that fail to meet the demands of the sport. Furthermore, psychological pressures related to performance and social influences, such as the dietary choices of teammates or trends observed on social media, can lead to eating behaviors that are not aligned with the specific needs of professional football. For adolescent and young players, nutritional deficiencies are often the result of a combination of factors specific to this stage of development. Eating habits acquired during childhood, often shaped by family environment and cultural customs, may not always meet the high energy demands of high-level sports. Additionally, these young athletes are particularly susceptible to the influence of social media, which frequently disseminates unvalidated nutritional information. The lack of regular follow-up by nutritionists or dietitians exacerbates this situation, as nutritional needs evolve rapidly during adolescence. The prevalence of fast food and processed foods, compounded by social constraints and a busy schedule, also contributes to an imbalance in macronutrients. Finally, inadequate meal planning, combined with often demanding training and school schedules, further limits young players’ ability to adhere to the nutritional recommendations necessary to support their growth and sports performance.

### 4.5 Limitation

The present study primarily focuses on analyzing players’ performance and nutritional status without considering their on-field positions. Therefore, one limitation is the lack of consideration for the effort exerted and the nutritional intake based on playing positions and the tactical schemes of the coaches. Ultimately, the main objective was to gain a general overview of the teams and various studied categories, rather than focusing on specific criteria related to players’ positions. Additionally, we did not account for cooking methods or the type of diet followed by the players. Our aim was to provide a general understanding of dietary intake without delving into these specific aspects. Future studies may consider these elements to better contextualize the Moroccan football landscape.

Despite these limitations, this study presents several strengths. It is one of the few studies that analyze the nutritional intake of Moroccan football players, providing valuable insights into their dietary habits in comparison with international recommendations (FIFA, UEFA). Additionally, the study takes into account a significant sample of players from different age categories, which offers a comprehensive overview of the nutritional practices across various levels of play. Moreover, the study's focus on traditional Moroccan meals, adapted for sports performance, brings a unique cultural perspective to the field of sports nutrition, contributing to a better understanding of how local dietary habits may impact performance in high-level athletes.

## 5 Conclusion

This study provides a comprehensive overview of dietary habits and their impact on the performance of Moroccan football players. It is evident that players exhibit shortcomings relative to nutritional recommendations, particularly regarding carbohydrate intake. Furthermore, a significant correlation has observed between protein intake and performance among young people and adolescents, highlighting the importance of this nutrient for athletic development. However, additional adjustments in diet may be necessary to fully meet recommendations. Regarding fat intake, an inverse relationship has observed with performance across all player categories. These findings underscore the crucial importance of a balanced diet tailored to players’ specific needs to maximize their athletic performance, as well as the necessity of preventive measures such as age-appropriate training programs and appropriate nutritional guidance.

For these reasons, we recommend several measures to improve the nutrition of professional and adult football players, as well as adolescents and young footballers. It is essential to ensure regular access to a varied and balanced diet, including fiber, vitamins, and essential fatty acids such as omega-3 and omega-6. Nutritional programs should incorporate specific meal planning tailored to the demands of training sessions and competitions. The implementation of personalized nutritional monitoring, conducted by qualified dietitians or nutritionists, is crucial for adjusting diets according to the individual needs of athletes. For young players, early nutritional education will be recommended to promote healthy eating habits and mitigate the impact of scientifically unfounded diets. Additionally, appropriate management of hydration and dietary intake must be ensured. Finally, it is important to raise awareness among players about the effects of psychological pressures and social influences on their dietary choices, while developing stress management strategies that preserve the quality of their nutrition.

## Data Availability

The original contributions presented in the study are included in the article/Supplementary Material, further inquiries can be directed to the corresponding author.
